# Is *Bifidobacterium breve *effective in the treatment of childhood constipation? Results from a pilot study

**DOI:** 10.1186/1475-2891-10-19

**Published:** 2011-02-23

**Authors:** MM Tabbers, I de Milliano, MG Roseboom, MA Benninga

**Affiliations:** 1 Department of Paediatric Gastroenterology and Nutrition, Emma Children's Hospital/Academic Medical Centre, Amsterdam, The Netherlands

## Abstract

**Background:**

Probiotics are increasingly used in the treatment of functional gastrointestinal disorders. Studies in constipated adults with a Bifidus yoghurt (containing *Bifidobacterium breve*, *Bifidobacterium bifidum *and *Lactobacillus acidophilus*) showed a significant increase in defecation frequency. The aim of this pilot study was to determine if *Bifidobacterium breve *is effective in the treatment of childhood constipation.

**Methods:**

Children, 3 to 16 years of age, with functional constipation according to the Rome III criteria were eligible for this study. During 4 weeks, children received one sachet of powder daily, containing 10^8^- 10^10 ^CFU *Bifidobacterium breve*. Furthermore, children were instructed to try to defecate on the toilet for 5-10 minutes after each meal and to complete a standardized bowel diary daily. The primary outcome measure was change in defecation frequency. Secondary outcome measures were stool consistency using the Bristol stool scale frequency of episodes of faecal incontinence, pain during defecation, frequency of abdominal pain, frequency of adverse effects (nausea, diarrhoea and bad taste), and frequency of intake of bisacodyl.

**Results:**

Twenty children (75% male, mean age 7.4) were included in this pilot study. The defecation frequency per week significantly increased from 0.9 (0-2) at baseline to 4.9 (0-21) in week 4 (p < 0.01). The mean stool consistency score increased from 2.6 (2-4) at baseline to 3.5 (1-6) in week 4 (p = 0.03). The number of faecal incontinence episodes per week significantly decreased from 9.0 (0-35) at baseline to 1.5 (0-7) in week 4 (p < 0.01). Abdominal pain episodes per week significantly decreased from 4.2 (0-7) at baseline to 1.9 (0-7) in week 4 (p = 0.01). No side effects occurred.

**Conclusion:**

*Bifidobacterium breve *is effective in increasing stool frequency in children with functional constipation. Furthermore it has a positive effect with respect to stool consistency, decreasing the number of faecal incontinence episodes and in diminishing abdominal pain. A randomized placebo controlled trial is required to confirm these data.

## Background

Functional constipation is a common and frustrating problem in childhood with an estimated prevalence of 3% in the western world [1]. This chronic condition is characterised by infrequent defecation less than three times per week, more than two episodes of faecal incontinence per week, the passage of large and painful stools which clog the toilet and retentive posturing. Upon physical examination a palpable faecal mass is often found in the abdomen and the rectum [2,3]. It causes distress to child and family and results in severe emotional disturbance and family discord [4]. The pathophysiology underlying functional constipation is undoubtedly multi-factorial, and not well understood. Withholding behaviour is probably the major cause for the development of constipation and might be caused by the previous production of a large, hard painful stool, anal fissures, a primarily behavioural mechanism or the resistance to go to another toilet then their own [4].

To date, patients are treated with a combination of education, toilet training and oral laxatives. Disappointingly, only 50% of all children followed for 6 to 12 months are found to recover and were successfully taken off laxatives [5]. Another study showed that despite intensive medical and behavioural therapy, 25% of patients developing constipation before the age of 5 years continued to have severe complaints of constipation beyond puberty [6]. Furthermore, in 50% of the patients using these compounds, adverse side-effects were registered such as: abdominal pain, bloating, flatulence, diarrhoea, nausea and bad taste [7]. No data exist concerning possible long-term adverse effects such as electrolyte disturbances, mucosal damage and habituation.

Probiotics are defined as live micro-organisms which when administered in adequate amounts, 10^6^-10^9 ^colony forming units, confer a health benefit on the host [8]. The use of probiotics has entered mainstream medicine and has proven being an effective therapy in many different gastrointestinal disorders, including functional gastrointestinal disorders [9,10]. However, large trials investigating the efficacy and safety of probiotics in pediatric patients are lacking [11,12]. Studies in constipated adults with a Bifidus yoghurt, containing *Bifidobacterium breve*, *Bifidobacterium bifidum *and *Lactobacillus acidophilus*, showed a significant increase in defecation frequency without any side effects [13,14]. Furthermore, a randomized controlled trial using *Bifidobacterium breve *in preterm infants showed less gas accumulation in the stomach, less vomiting and improved weight gain without any side effects, suggesting a positive effect on gastrointestinal motility [15] The exact working mechanism of probiotics are not well understood. There are some hypotheses, however, why probiotics might have therapeutic potential for the treatment of constipation. Firstly, a dysbiosis in the gut flora in constipated patients has been suggested which might improve after the ingestion of probiotics [9,10]. It remains important however to understand if dysbiosis is a secondary manifestation of constipation, or if it is a factor contributing to constipation. Furthermore, probiotics can lower pH of the colon by producing lactic, acetic and other short chain fatty acids. A lower pH enhances colonic peristalsis and subsequently decreases colonic transit time [9,10].

Based on the positive data in constipated adults, we therefore performed a pilot study to determine if *Bifidobacterium breve *is effective in the treatment of children with constipation.

## Methods

### Subjects

Children, 3 to 16 years of age, referred to the outpatient clinic of a tertiary pediatric gastroenterology department, with constipation were eligible for this study. Patients were included if they had been suffering from functional constipation according to the Rome III criteria for the last 2 months [2,3]. All children included had a defecation frequency of <3 times/week and one or more of the following criteria: faecal incontinence >1 episode/week, a large amount of stools that clog the toilet, painful defecation, withholding behaviour, or abdominal or rectal faecal impaction upon physical examination. In order to obtain a homogeneous group of patients, we included children with a defecation frequency <3 times/week in combination with at least one other ROME III criterion. Patients were not enrolled in this study if they had been treated for constipation less than 2 weeks before the start of the study. Other exclusion criteria were: a diagnosis of either IBS or functional non-retentive faecal incontinence according to the Rome III criteria; a diagnosis of mental retardation or metabolic disease (hypothyroidism), Hirschsprung's disease, spinal anomalies, anorectal pathology, previous gastrointestinal surgery. All children older than 12 years and/or parents gave informed consent. This pilot was approved by the medical ethical committee,

### Study design

Seven days prior to baseline assessment and during the treatment period, all children recorded frequency of bowel movements, stool consistency according to the Bristol stool scale, the number of faecal incontinence episodes, pain during defecation, abdominal pain, as well as adverse effects such as vomiting and diarrhoea in a standardized bowel diary. At baseline a medical history and information on the current defection pattern was collected and also a physical examination including a rectal digital exam was performed. Information and education about functional constipation was given to all patients and their care takers. Before start of the probiotic treatment, all children received once daily for 3 days a rectal enema in order to accomplish rectal disimpaction. After rectal disimpaction, all children received daily one sachet of powder containing 10^8-10 ^CFU *Bifidobacterium breve *Yakult, for 4 weeks. The patients were allowed to mix the powder with all liquids on condition that the liquid was not hot. During the study, all children were instructed to try to defecate on the toilet for 5-10 minutes after each meal (3 times a day). Patients were not allowed to consume any other fermented dairy product during this study or any other laxatives, except for the rescue medication Biscaodyl. Names of the forbidden products were pointed out in the diary. During the product consumption period, patients were instructed to take bisacodyl 5 mg if they did not defecate for 3 consecutive days. Clinical evaluation, frequency of adverse effects and compliance to the study protocol were carried out at enrolment and at 2, 4 and 6 weeks using standardized bowel diaries.

### Outcome measures

The primary outcome measure was change in defecation frequency in week 4 compared to baseline. Secondary outcome measures were stool consistency, frequency of episodes of faecal incontinence, pain during defecation, frequency of abdominal pain, frequency of adverse effects (nausea, diarrhoea and bad taste), and frequency of intake of bisacodyl.

### Analysis

Descriptive statistics were performed for baseline characteristics, adverse effects and Bisacodyl use. Change of frequency of bowel movements, fecal incontinence and change of stool consistency, was assessed using the non-parametric paired Wilcoxon test. For the comparison of abdominal pain between baseline and the evaluation time points, the Wilcoxon rank test was used. A p- value < 0.05 was considered to be significant. All analyses were done with SPSS (version 16.0).

## Results

Between July 2009 and January 2010, 22 children were included into this pilot study. Two children were lost to follow-up without having any outcome data during follow-up. Both patients were therefore excluded from the final analysis. All outcomes of the remaining 20 children were collected and analyzed. The baseline characteristics are described in Table 1 (mean (range))

**Table 1:** Baseline patient characteristics: mean (range)

Age in years	7.4 (4-13)
Boys, n (%)	15 (75%)
Mean stool frequency per week	0.9 (0-2)
Stool consistency score	2.6 (2-4)
Faecal incontinence episodes per week	9 (0-35)
Pain during defecation	71% (12/17)
Abdominal pain episodes per week	4.2 (0-7)

**Figure 1:**
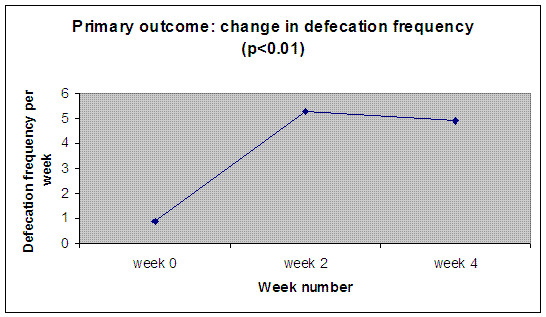
**Primary outcome: change in defecation frequency (n = 20)**. X-axis: week number. Y-axis: defecation frequency per week

The defecation frequency per week significantly increased from 0.9 (0-2) at baseline to 5.3 (0-21) in week 2 (p < 0.01) and 4.9 (0-21) in week 4 (p < 0.01) (figure 1: Defecation frequency over 4 weeks.). The mean stool consistency score significantly increased from 2.6 (2-4) at baseline to 3.6 (1-6) in week 2 (p = 0.07) and 3.5 (1-6) in week 4 (p = 0.03). The episodes of faecal incontinence per week significantly decreased from 9.0 (0-35) at baseline to 2.6 (0-7) in week 2 (p = 0.01) and 1.5 (0-7) in week 4 (p < 0.01). Pain during defecation decreased from 71% (12/17) at baseline to 40% (8/20) in week 2 (p = 0.10) and 33% (6/18) in week 4 (p = 0.08). Abdominal pain episodes per week significantly decreased from 4.2 (0-7) at baseline to 2.2 (0-7) in week 2 (p = 0.02) and 1.9 (0-7) in week 4 (p = 0.01). Bisacodyl was used by 45% of patients during week 1, 25% of patients during week 2, 35% of patients during week 3 and by 20% of patients during week 4. No side effects were reported such as nausea, diarrhoea and bad taste or increased flatulence during the study period. Table 2 gives an overview of the outcome measures with p-values.

**Table 2:** Main outcome measures (mean) with p-values (n = 20)

Outcome	At baseline	Week 2	Week 4	P-value (week 4-week 0)
Defecation frequency p/week	0.9	5.3	4.9	p < 0.01

Stool consistency score	2.6	3.6	3.5	p = 0.03

Episodes of faecal incontinence p/week	9.0	2.6	1.5	p < 0.01

Pain during defecation	71%	40%	33%	p = 0.08

Abdominal pain episodes p/week	4.2	2.2	1.9	p = 0.01

## Discussion

This pilot study showed that intake of *Bifidobacterium breve *for 4 weeks, significantly increased the defecation frequency. Furthermore, stool consistency, the frequency of episodes of faecal incontinence and the frequency of abdominal pain significantly changed after the use of this specific probiotic strain.

A recent systematic review on the effects of laxative treatment and dietary measures in the management of childhood constipation found only 2 randomized controlled trials evaluating the effects of probiotics [16]. In the first small study, 45 children younger than 10 years with chronic constipation were randomly assigned to receive magnesium oxide (50 mg/kg/day (n = 18), or 8 × 10^8 ^cfu/day of the probiotic *Lactobacillus casei rhamnosus *(n = 18), or placebo (n = 9) twice daily for 4 weeks [17]. No statistically significant difference in the defecation frequency per day was found between the probiotic group and the magnesium oxide group. However, patients receiving either the probiotic strain or the oral laxative had a significantly higher defecation frequency compared to the placebo group (defecation frequency [times/day 0.57 ± 0.17 and 0.55 ± 0.13, respectively, compared to 0.37 ± 0.10, p = 0.03). The second trial was conducted to determine if *Lactobacillus rhamnosus *GG (LGG) is an effective adjunct to lactulose for treating constipation in children. A total of 48 children with constipation received 1 ml/kg/day of 70% lactulose plus 10^9 ^cfu of LGG or 1 ml/kg/day of 70% lactulose plus placebo, twice daily for 12 weeks [12]. There were no significant differences in rates of product success (defined as ≥ 3 spontaneous stools per week with no faecal incontinence) at 12 and 24 weeks between the LGG group (rates: 72% and 64%, respectively) and the placebo group (rates: 68% and 65%, respectively). `

In a recent trial, 44 children, at least 6 months old, with chronic constipation were randomly assigned to receive supplementation with the probiotic *Lactobacillus reuteri *(DSM 17938) (n = 22) or placebo (n = 22) [18]. Infants receiving *Lactobacillus reuteri *had a significantly higher frequency of bowel movements than infants receiving a placebo at week 8 of supplementation (2.82 per week at week 0, compared with 4.77 at week 8 in the probiotic group, absolute numbers not given for placebo group, P = 0.027). There was no significant difference between *Lactobacillus reuteri *and placebo groups in the stool consistency at all weeks nor in the presence of inconsolable crying episodes. All three trials did not report any adverse events in the probiotic group.

A mixture of probiotics (containing *Bifidobacteria (B.) bifidum*, *B. infantis*, *B. longum*, *Lactobacillus (L.) casei*, *L. plantarum *and *L. rhamnosus*), increased the number of bowel movements, decreased the number of faecal incontinence episodes and improved the consistency of stools [19]. Although the results of this small pilot study are positive, a large randomized controlled trial is necessary to confirm these data.

It is unknown why the outcome of the above mentioned trials in constipated children is so different. We speculate that the use of different inclusion criteria for paediatric constipation with consequently different study populations and the use of different probiotic strains, given in different dosages with variable duration of the treatment period, influence study outcomes.

In contrast to the few paediatric studies, data suggest that adults with constipation might benefit from ingestion of *B. lactis *DN-173 010, *L. casei *Shirota, and *E. coli *Nissle 1917. All studies showed an increased defecation frequency and improved stool consistency [20]. These findings, however are not directly applicable to the paediatric population because constipation in children differs considerably from that in constipated adults with regard to its prevalence, onset, aetiology, symptoms, treatment, and prognosis [21].

Besides the increase in defecation frequency and decrease in episodes of faecal incontinence, this study also showed a significant effect in softening of stools and in decreasing abdominal pain. Both effects could be a direct consequence an increase in defecation but theoretically, it could also be caused by the working mechanism of the probiotics. It has been assumed that probiotics soften the stools by stimulating water and electrolyte secretion [22,23]. Furthermore, one paediatric study and several studies in adults with irritable bowel syndrome (IBS), have demonstrated that abdominal pain decreased when using probiotics [17,24,25]. Whorwell et al. conducted a randomized trial in 360 women with IBS receiving *Bifidobacterium infantis and *found a significant improvement of abdominal pain which occurred irrespective of any effect on stool frequency. The authors hypothesized that the probiotics were able to diminish visceral hypersensitivity by its anti-inflammatory effect on the enteric mucosa [26].

According to the available data, it is assumed that the risk of infection with the probiotic lactobacilli or bifidobacteria is similar to risks with commensal strains [10]. However, there is concern that the use of probiotics may result in harmful events in at-risk populations like immunocompromized subjects or in patients with other life-threatening illnesses, who were admitted in the intensive care unit. Based on our results and their safety profile, adding probiotics to the standard treatment of functional constipation in otherwise healthy children is promising. The major limitation of our study is that this study is a non randomized non placebo controlled small pilot study. However, since this study shows promising results it is worthwhile to perform a large RCT to unravel the efficacy of *Bifidobacterium breve *in constipated children.

In conclusion, this small pilot study suggests that *Bifidobacterium breve *is effective in the treatment of childhood constipation. A large randomized placebo controlled trial is now required to confirm these data.

## Competing interests

The authors declare that they have no competing interests.

## Authors' contributions

MMT and MAB participated in the design of the study

All authors collected the data.

MMT and IDM did the statistical analysis

MMT drafted the first manuscript

All authors read and approved the final manuscript
